# Mechanical behavior of implant assisted removable partial denture for Kennedy class II

**DOI:** 10.4317/jced.56533

**Published:** 2020-01-01

**Authors:** João-Paulo-Mendes Tribst, Rodrigo-Máximo de Araújo, Naiara-Pires Ramanzine, Natália-Ribeiro Santos, Amanda-Maria-de Oliveira Dal Piva, Alexandre-Luiz-Souto Borges, João-Mauricio-Ferraz da Silva

**Affiliations:** 1 DDs, MSc, PhD Student, Department of Dental Materials and Proshodontics, São Paulo State University (Unesp), Institute of Science and Technology, São José dos Campos / SP, Brazil; 2 DDs, MSc, Professor, Department of Dental Materials and Proshodontics, São Paulo State University (Unesp), Institute of Science and Technology, São José dos Campos / SP, Brazil; 3 DDs, São Paulo State University (Unesp), Institute of Science and Technology, São José dos Campos / SP, Brazil

## Abstract

**Background:**

This study evaluated the mechanical response of a removable partial denture (RPD) in Kennedy Class II according to being associated or not with implants.

**Material and Methods:**

Four RPDs were manufactured for a Kennedy Class II: CRPD - Conventional RPD, RPD+1M, RPD+2M and RPD+12M, respectively, signifying implant assisted RPDs with the implant installed in the first molar, second molar, and in the first and second molars. The finite element method was used to determine the most damaged support tooth under compressive load (300N, 10s) and strain gauge analysis was used to evaluate the microstrain. All groups were submitted to a retentive force analysis (0.5 mm/mm, 100kgf). Microstrain and retentive force data were submitted to One-way ANOVA and the Tukey test, all with α=5%.

**Results:**

High microstrain was observed in the second premolar adjacent to the edentulous space under compression load (*p*< 0.01). RPD+12M presented lower microstrain, however being similar to RPD+2M. RPD+1M presented a higher mean value of retentive force, but similar to RPD+12M. FEM showed RPD assisted by implants concentrates less stress in the periodontal ligament. The association of two implants was sufficient to decrease the stress generated in the implants. The most stressed region for the o-ring abutment was the threads, and the group with two implants showed the lowest stress concentration.

**Conclusions:**

In cases of Kennedy Class II, the association of RPD with implants in the molar region is a favorable option for patient rehabilitation, reducing the movement of the direct retainer adjacent to the edentulous space, increasing the removal force and decreasing the stress magnitude in the periodontal ligament.

** Key words:**Removable partial denture, Finite element analysis, Prosthetic dentistry, Implant-assisted RPD, Distal extension RPD considerations.

## Introduction

The total loss of teeth has decreased significantly with the advance in preventive dentistry, whereas the number of partially-toothed patients has increased (1). A removable partial denture (RPD) is an alternative treatment to fixed prostheses in teeth or implants (2). However, in situations where the posterior tooth support is absent (Kennedy classes I and II), the success rate is lower compared to situations with dental support (classes III or IV) (2). Distal extension RPDs are subjected to different forces (vertical, horizontal, torque) which compromise the stability and prosthesis retention (3). Prosthesis distal rotation (4) acts as a fulcrum, creating a levering motion and compressing the soft tissues, generating displacement in the distal extension RPD (5). This movement results in unfavorable horizontal forces, facilitating unwanted bone remodeling and possible loss of the supporting teeth (6). To avoid these disadvantages, the association of RPD with osseointegrated implants in the edentulous region emerged to improve the biomechanical behavior of removable distal extension partial dentures (2,5-10). An osseointegrated dental implant in the posterior edentulous region distal to the terminal abutment provides improved vertical support to the RPD distal extension (6). Implant-assisted RPDs (IARPD) aim to provide comfort, support, retention, stability and better prognosis for the abutment teeth (2,9-11). Implant installation creates a dental support condition which minimizes the lever observed in the distal abutments during function (2).

Some studies which evaluated the mechanical response of the IARPD used stress and strain analysis with the finite element method (5,10-14). However, validation of the numerical model is not always emphasized in these studies. The results usually do not demonstrate the effect generated in the implant and abutment platform (10,12-14), while some studies are limited to only one criteria of analysis, usually von-Mises stress (5,13). Therefore, questions arise about how to select the number and position of the implants in order to promote adequate biomechanical performance of the IARPD.

In view of the above, the present study aimed to evaluate the stress distribution, strain and displacement of RPD in unilateral posterior edentulous space *in vitro* and in silico according to an association or not with implants. The hypothesis of the study consisted that the number of implants (1 or 2) and location (first molar and/or second molar region) would not influence the mechanical response of RPDs.

## Material and Methods

-Specimen preparation 

**Figure 1:**
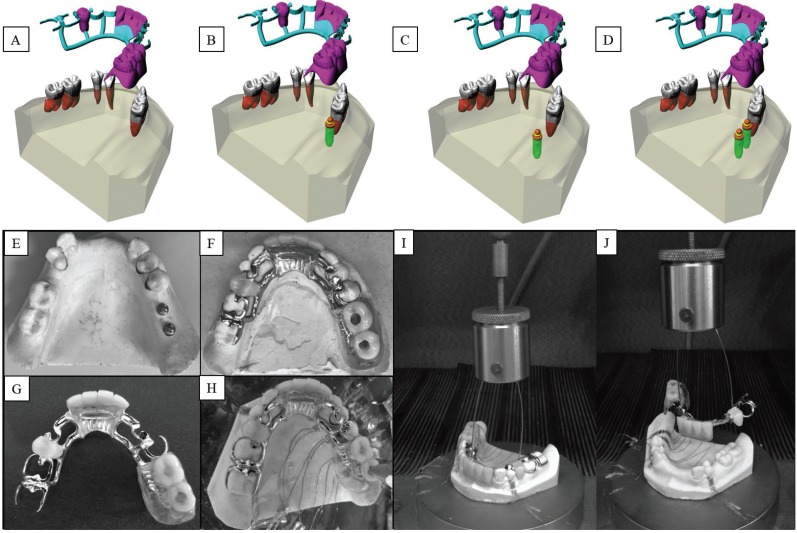
(A-J) - Schematic illustration of the 3D modeling using in this study. The groups were divided in (A) CPRD - Conventional RPD, (B) RPD+1M: Implant assisted RPD with the implant installed in the first molar region; (C) RPD+2M: Implant assisted RPD with the implant installed in the second molar region; and (D) RPD+12M: Implant assisted RPD with the implants installed in the first and second molar regions. Figure 2A-C shows A RPD+12M, the prosthesis in position and the RPD itself. (E-J) Representative specimen submitted to the compression load. E) Polyurethane model of Kennedy Class II assisted by two implants (RPD+12M). F, G) Final implant assisted RPD. H) Strain gauges glued to the polyurethane model and submitted to the compression load in the second molar region. I, J) Specimen submitted to the *in vitro* testing.

A stone model of an arch Class II of Kennedy modification 2 was duplicated using vinylpolysiloxane impression material (Elite H-D Putty and Elite H-D Light Body, Zhermack). Next, a model of polyurethane resin (F160-resin Axson Brasil Industria e Comercio Ltda) was made by mixing part A (Polyol) with part B (Isocyanate), following the recommended mixing ratio of 1:1 by weight, which was handled in a plastic container with plastic spatula (15,16). The polyurethane resin model represented Class II of Kennedy modification 2, with its direct and indirect retainers in typodont. A fluid elastomer positioned between teeth and alveolus (Flexitime Easy Putty; President Light Body; Xantopren L Blue) was used to simulate the periodontal ligament. Afterwards, this model was designed to determine an insertion path for the future RPD by the Applegate method (17). Thereafter, the guide plane preparations and rests were performed on the support teeth. The model was then impressed with condensation silicone in two stages after the preparation. First a pre-molding with the dense silicone (Flexitime Easy Putty; President Heavy Putty; Optosil), where a relief was inserted throughout the mold and then the impression with the fluid silicone (Flexitime Easy Putty; President Light Body; Xantopren). The impression was used to obtain the stone model (Elite Base, Zhermack) which was sent to a dental technician to manufacture the metal frame. The metal frame was tested in the model and returned to the dental technician to manufacture an acrylic resin base in its free end region. These procedures were repeated to obtain four different groups: CPRD - Conventional RPD, RPD+1M: IARPD with the implant installed in the first molar region; RPD+2M: IARPD with the implant installed in the second molar region; and RPD+12M: IARPD with the implants installed in the first and second molars region. Figure 1(A-D) summarizes the group distribution. The manufacturer’s drilling protocol (Conexão Prosthesis Systems, São Paulo, Brazil) was used for the implant installation, respecting the length and diameter of the selected implants (3.75 x 10 mm). The implants were installed with the aid of a manual wrench with a torque of 40 N.Cm. An O-ring type component was installed on the implants with a torque of 35 N.Cm, according to the manufacturer’s recommendation. The cylinders were subsequently positioned on the abutments. In the groups containing implants, the RPDs were relieved in the region corresponding to the cylinder until their final settlement position. Acrylic resin (Duralay ™, Reliance Dental Manufacturing) was used to attach the cylinder to the base of the prosthesis using the liquid-powder technique.

-Compression load

The surfaces of the polyurethane models were carefully cleaned with isopropyl alcohol. Next, ten linear strain gauges (KFG-02-120-C1-11, Kyowa Eletronic Instruments Co.) were glued with cyanoacrylate based adhesive (SuperBonderLoctite, São Paulo, Brazil) on the buccal and lingual bone regions from the implant and positioned equidistant around teeth 34, 44 and 45, respectively, constituting direct and indirect retainers of the partially edentulous arches used in the study (Fig. 1H). Each strain gauge was then measured using a multimeter device (Minida ET 2055: Minida) (15). The assembly was tested in a universal testing machine (DL-1000, EMIC, São josé dos *Pi*nhais, Brazil) to apply a compressive load (1000N load cell and 2 mm/min crosshead speed) until a maximum load of 300N which remained for 10 s. The 3 mm diameter loading tip was positioned at the center of the occlusal face of the second molar of the RPD (15). Variations of electrical resistance were converted to microstrain units through an electrical signal conditioning apparatus (Model 5100B Scanner, Instruments Division Measurements Group, Inc. Raleigh). Electrical cables enabled the connection between the strain gauges and the data acquisition apparatus in which the data reading was performed (StrainSmart® Data Acquisition Software, Micro-Measurements) (15,18). This analysis was used to determine which region would present the highest microstrain values in the bone simulator. Figure 1 (E-J) summarizes the *in vitro* specimen and testing.

-Removal force

The polyurethane model was fixed with cyanoacrylate based adhesive on the testing platform of the universal testing machine to perform the removal force analysis of each group. The RPD was then positioned. The test was performed with 0.5 mm/mm through orthodontic wire number 0.7 and load cell of 100 kgf (19) (Fig. 1I,J). Twenty repetitions were performed for each prosthesis and the data was obtained in Newton (19). This test was used to determine the necessary force to remove the prosthesis.

-Finite element analysis

The analysis of the stress distribution in the teeth, implants and periodontal ligaments was performed using the finite element method (FEM). To do so, the model of the polyurethane mandible used in the *in vitro* test was scanned using an intraoral scanner (CEREC AC Omnicam, Sirona). The three-dimensional (3D) STL file was imported to the modeling software (Rhinoceros 4.0 SR9, McNeil). Anatomical lines of the mesh surface were created by applying the BioCad protocol (20). With the determined lines, the surfaces and solids were closed for a volumetric 3D model similar to the *in vitro* one. The root surface of each tooth received an offset expansion of 0.3 mm for the creation of the periodontal ligament within the alveolus (21). Next, the first part to conduct the RPD modeling was to create the framework. To do this, the metallic structure was delimited with lines following the *in vitro* position. Next, the major connector, minor connectors and saddle received the command pipe to create a volumetric cylindrical shape. The clasps were subsequently modeled with the same dimensions of the *in vitro* model in the same position. A Boolean union was used between clasps and frameworks. The acrylic resin was created to replace the loose tooth. The set RPD and mandible were replicated in four models according to the groups’ distribution, CRPD, RPD + 1M, RPD + 2M and RPD +12M (Fig. 1). For the three IARPDs, an external hexagon implant (3.75 x 10 mm) was placed in the first molar, second molar and both regions, respectively. The O-ring abutments were modeled containing a rubber ring and titanium cylinder at the top. The Boolean union was used between titanium cylinder and the acrylic resin of the prostheses. After modeling, the geometries were exported in STEP format to the analysis software (ANSYS 17.2, ANSYS Inc.) where they were subdivided in a finite element mesh. The number of nodes and elements were controlled by a mesh convergence test with the maximum element size of 0.3 mm and the aspect ratio of 1.56. The contacts were considered ideal, the fixation was defined in the base of the model and the load was applied with the same parameter as the *in vitro* test (300 N, 6 mm in the occlusal surface of the second molar) (15). The material properties used in the simulation were obtained from the literature and are summarized in  Table 1 (22-25). The results were required in von-Mises stress distribution for all sets, maximum principal stress for the teeth, maximum principal strain for the periodontal ligament and directional deformation (y-axis) for the second premolar adjacent to the edentulous area.

**Table 1:**
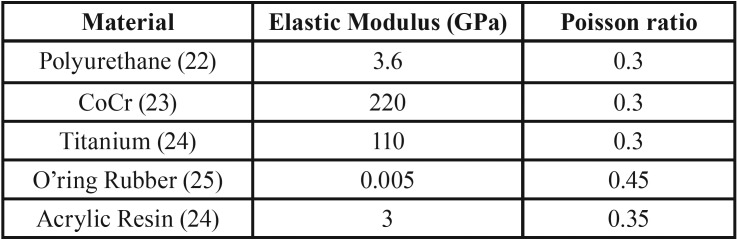
Material mechanical properties used in the computational simulation.

-Data analysis

The microstrain results were analyzed by descriptive statistics (means and standard deviation) and used for subsequent comparison with the values obtained in the numerical simulation (18). Next, the maximum microstrain and removal load values were evaluated using one-way analysis of variance (ANOVA) followed by the Tukey test, all with α = 5%, using a statistical software program (Minitab 17, Minitab Inc.). The FEM results were plotted in stress maps and the maximum stress values were plotted in  Table 2.

**Table 2:**
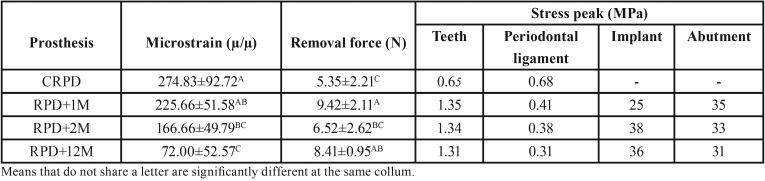
Descriptive statistical analysis (means ± standard deviation) and Tukey test (α = 5%) according to the evaluated prosthesis.

## Results

-Compression load

**Figure 2:**
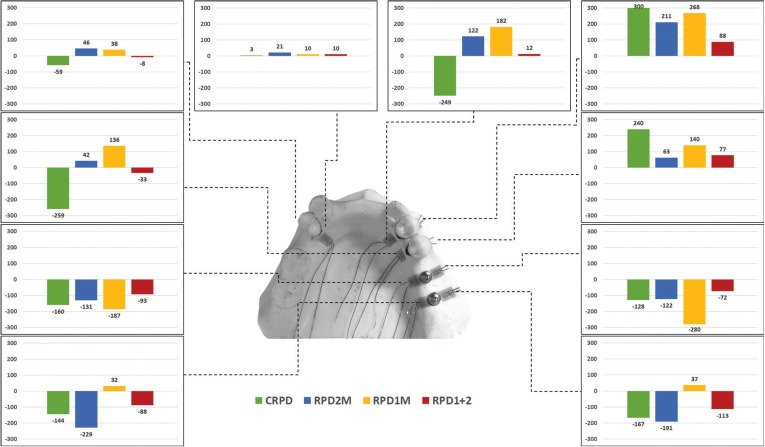
Microstrain analysis in the bone tissue according to the prosthesis: CPRD, RPD+1M, RPD+2M and RPD+1 and 2M.

By analyzing the generated microstrain in the evaluated abutments (buccal and lingual) (Fig. 2), it was observed that the maximum microstrain occurred in the second premolar adjacent to the edentulous space. It was qualitatively observed that the second premolar as a direct retainer presented less movement when the RPD was assisted by implants. In relation to the first premolar (splinted to the second premolar), similar behavior was observed for the movement generated in relation to the position and number of implants associated with PRPD, but with a lower magnitude. For tooth 34, an indirect retainer, it was found that the presence or absence of implants in the free end region did not interfere with the generated torque. One-way ANOVA showed that the microstrain values generated in abutment 44 was statistically influenced by the prosthesis (*p* < 0.001). The Tukey test showed that the use of two implants (RPD+12M) presented a lower mean microstrain value, however it was statistically similar to the RPD+2M condition.

-Removal force

Mean values for removal force were also affected by the type of prosthesis, according to one-way ANOVA (*p* < 0.001). The Tukey test showed that the use of an implant in the first molar region presented a higher mean value of load for removal, however similar to the mean load of the two implant group.

-Finite element analysis

**Figure 3:**
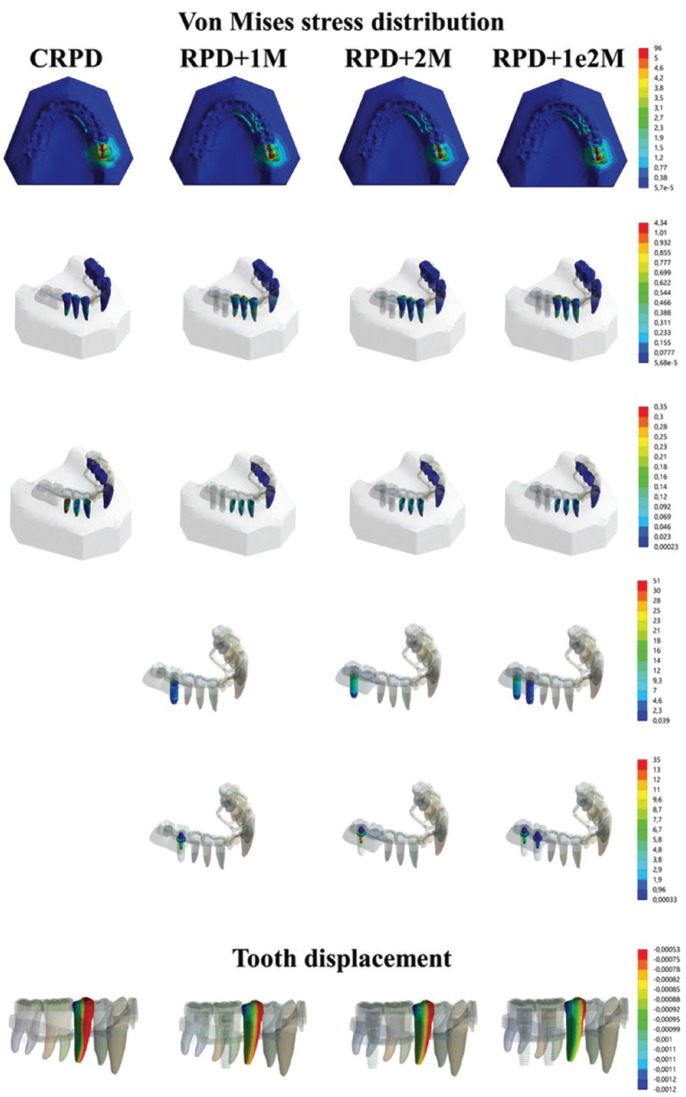
Von Mises stress distribution in the set, teeth, periodontal ligament, implant and abutment, and tooth displacement.

FEM (Fig. 3) presented the same mechanical behavior calculated *in vitro* for the movement generated in the second right lower premolar. Thus, the mathematical model was assumed to be valid for anatomical structures and dental implants. In observing the stress maps, it is possible to observe that the stress generated in the masticatory load region generally presents the same pattern for all groups. Moreover, the stress generated in the remaining teeth demonstrated that there is less accumulated stress in the root of the supporting teeth when these are not assisted by implants. However, the von Mises generated in the periodontal ligament shows that the worst scenario is observed for the conventional prosthesis (CRPD). A higher stress concentration was observed in the RPD+2M group in observing the von Mises stress generated in the implants, as its implant is exactly under the region of the load application, followed by the RPD+12M group for the same distal implant, but with a lower magnitude. The RPD+1M group presented less stress in its implant structure; however this was lower for the implant in that same region for the RPD+12M group. Therefore, the association of two implants was sufficient to decrease the stress generated in the implants compared to unitary implants. The stress generated in the prosthetic components was selected to demonstrate possible damage in the structures responsible for connecting the prosthesis to the implant. The most stressed region of the abutment were the wires, and the group with two implants showed the lowest stress concentration.

## Discussion

This study evaluated the stress distribution, microstrain and displacement *in vitro* and *in silico* of a conventional RPD for unilateral posterior edentulous space and the same prosthesis in three situations in association with dental implants. The results demonstrated that there is a modification in the mechanical response generated with the association of one or two implants, as well as their installation position. Thus, the null hypothesis was denied.

The results of the present study corroborate the literature that the association of implants and RPDs improves the biomechanical properties of the prosthesis (2,10). Verri *et al.* (12) used the two-dimensional finite element method and concluded that the association of an implant with RPD provided more support to the base of the RPD, reducing its intrusion on the fibromucosa. The findings observed in this study corroborate these results in the sense of the mechanical benefit due to the presence of the implant assistance. However, the evaluation with three-dimensional and *in vitro* models measuring the stresses generated in the abutments, implants, periodontal ligament and teeth more precisely show which structures were benefited or not by the association between implants and the RPD. Moreover, unlike the results found in this study, the authors report that the presence of the osseointegrated implant did not favor a reduction in the stress concentration in the supporting tooth of the RPD, adjacent to the edentulous space, which may have occurred due to simplifying the bi-dimensional model. Regarding benefits of the association of RPD with implants, it is possible to consider: the minimum number of implants which can be used, the remaining teeth are able to maintain the proprioception, the treatment cost can decrease, easier hygiene, and the patient’s expectations can be achieved (7). Moreover, the present study demonstrated that the implant association increases the retention force of the prosthesis, decreasing the movement of the direct retainers and enabling better dissipation of the masticatory loads.

The literature is fairly concise in reporting the mechanical benefit with finite element studies for Kennedy Class I (10,13,14,26-28), with few papers evaluating a unilateral situation (which is quite common), known as Kennedy’s Class II (1,2,7).

A study by Matsudate *et al.* (29) stands out because the effect of implant location on load distribution in the abutment tooth for Kennedy Class I IAPRD was evaluated using piezoelectric force transducers. Our findings corroborated these authors’ results in that the use of a distal implant is more beneficial than a mesial implant. The principal novelty in comparing the study of Matsudate *et al.* (29) with this study is the indication that using two implants is even more beneficial than a single distal implant, and that the observation of the stress results for the O-ring abutment, periodontal ligament and bone can enable clinicians to perform this indication.

Campos *et al.* (30) evaluated the oral health-related quality of life comparing the use of CRPD and IARPDs. The authors showed a reduction of 100% in discomfort and 80% in pronunciation problems when a posterior implant was installed. Our results corroborate those findings, since the removal force of the RPD increased with the implant placement, which means better stability in chewing and talking.

Comparing the levels of patient satisfaction with either conventional mandibular bilateral distal extension partial dentures or those assisted by bilateral distal implants (6), it is possible to note that there were significantly improved parameters of stability, chewing and overall satisfaction. This effect is even higher when the authors replaced the healing caps for O-ring abutments (27). In a literature review comparing retention systems, Trakas *et al.* (31) defined that the O-ring seems to convey minimal stress to the implants, thereby allowing adequate hygiene. For this reason, an O-ring abutment was simulated in the present study.

A previous paper analyzed the effect of the occlusal rest position on the IARPD by finite element analysis (14). The authors also simulated an O-ring attachment in the abutment, and reported that the rest position can modify the stress at the resin base. However, the information of stress concentration in the implants, abutment, tooth and periodontal ligament are missing. Mitrani *et al.* (32) reported that an RPD associated with an implant presents less bone remodeling under the prosthesis. At the same time, greater retention, stability and better function were observed in this type of implant assisted prosthesis. This can be explained by the results of bone microstrain observed in the present study, since the bone region under the RPD presents higher microstrain values when implants are installed, which represents greater mechanical stimulus in the same region.

Considering the results for the CRPD, tooth 44 also suffered movement during the saddle intrusion, as well as tooth 45. This is justified due to the splinting effect that is indicated for dental abutments, which is considered insufficient to retain the prosthesis alone (2,17). Even though the shape, root length and support bone tissue appear to be suiTable for a common abutment, the fact that the tooth does not have proximal contact endangers it, especially when it is used to sustain a free extremity RPD. The mathematical results in the periodontal ligament can be explained by the movement of the second right lower premolar, which is higher in the CPRD, followed by RPD+1M, RPD +2M and RPD +12M.

The effect of implant location on the stress distribution in distal extension IARPD has already been evaluated (13). The authors simulated a bilateral edentulous jaw, with unitary implant in second premolar region, first molar region or second molar region and then, a bilateral load was applied in the metal framework of the prosthesis. The authors concluded that the best position for implant positioning would be the first molar region. This is different from our indication that the best position for implant placement is in both missing teeth regions (RPD+12M), and if this is not possible, the next surgical approach should be to place an implant in the second molar region to reduce the cantilever effect, showing similar microstrain and removal force as if two implants were used. It is possible to justify this indication due to the periodontal ligament behavior and the movement of the sound direct retainer (second premolar) which are more important than the stress generated in the implant. This affirmation is based on the yield strength of titanium (more than 600 MPa) (33), and herein by the maximum stress peaks calculated for the implant which was 51 MPa and 35 MPa in the abutment. However, the continued lateral movement for the periodontal ligament and tooth can induce bone remodeling and insertion loss.

A finite element analysis of different implant positions was analyzed using a 3D FEM (10). Placing the implant in the first molar area resulted in improved displacement values, and reduced maximum stress values at the peri-implant bone area, metal structure, and implant were observed. However, the authors applied a load with 30 degrees of inclination in relation to the occlusal plane in all occlusal surfaces of all mandibular teeth at the same time. The authors considered a Kennedy Class I, with symmetric bilateral edentulous jaw and symmetric implants positioned in both sides. This can explain the difference between the results herein and their results, but those authors did not explain the benefits of two adjacent implants.

Some prosthetic complications can be observed even after the prosthesis finishing regarding relining, pitting of the healing abutment, replacement of the resilient component of the attachment, damage in the framework, screw loosening or damage in the acrylic denture base (28). Thus, control and continuous observation by the clinician is important. In addition, some study limitations such as the absence of temperature, pH, humidity and different loading during the simulation should be considered before comparison with other investigations.

Based on the results, it can be concluded that an association of RPD with osseointegrated implants in the molar region in cases of unilateral free-end is a favorable option for patient rehabilitation by reducing the movement of the direct retainer adjacent to the edentulous space, increasing the prosthesis removal force and decreasing the microstrain magnitude on the remaining periodontal ligaments in the teeth.

## References

[1] Douglass CW, Watson AJ (2002). Future needs for fixed and removable partial dentures in the United States. J Prosthet Dent.

[2] Kim JJ (2019). Revisiting the Removable Partial Denture. Dent Clin North Am.

[3] Krol A, Jacobsen T, Finzen F (1990). Removable Partial Denture Design. 4th ed.

[4] Phoenix RD, Cagna DR, De Freest CF (2008). Stewart's clinical removable partial prosthodontics. 4th ed.

[5] Eom JW, Lim YJ, Kim MJ, Kwon HB (2017). Three-dimensional finite element analysis of implant-assisted removable partial dentures. J Prosthet Dent.

[6] Ramchandran A, Agrawal KK, Chand P, Ramashanker, Singh RD, Gupta A (2016). Implant-assisted removable partial denture: An approach to switch Kennedy Class I to Kennedy Class III. J Indian Prosthodont Soc.

[7] Carvalho WR, Barboza EP, Caúla AL (2001). Implant retained removable prosthesis with ball attachments in partially edentulous maxilla. Implant Dent.

[8] Mijiritsky E, Karas S (2004). Removable partial denture design involving teeth and implants as an alternative to unsuccessful fixed implant therapy: a case report. Implant Dent.

[9] Roh KW, Jeon YC, Jeong CM, Yoon MJ, Lee SH, Huh JB (2018). Implant assisted removable partial denture with implant surveyed crown: A 20-month follow-up case report. J Korean Acad Prosthodont.

[10] Ortiz-Puigpelat O, Lázaro-Abdulkarim A, de Medrano-Reñé JM, Gargallo-Albiol J, Cabratosa-Termes J, Hernández-Alfaro F (2019). Influence of implant position in implant-assisted removable partial denture: a three-dimensional finite element analysis. J Prosthodont.

[11] Kuzmanovic DV, Payne AG, Purton DG (2004). Distal implants to modify the Kennedy classification of a removable partial denture: a clinical report. J Prosthet Dent.

[12] Verri FR, Pellizzer EP, Rocha EP, Pereira JA (2007). Influence of length and diameter of implants associated with distal extension removable partial dentures. Impl Dent.

[13] Memari Y, Geramy A, Fayaz A, Rezvani Habib Abadi S, Mansouri Y (2014). Influence of implant position on stress distribution in implant-assisted distal extension removable partial dentures: A 3D finite element analysis. J Dent (Tehran).

[14] Shahmiri R, Das R, Aarts JM, Bennani V (2014). Finite element analysis of an implant-assisted removable partial denture during bilateral loading: occlusal rests position. J Prosthet Dent.

[15] Tribst JPM, Dal Piva AMO, Riquieri H, Nishioka RS, Bottino MA, Rodrigues VA (2019). Monolithic zirconia crown does not increase the peri-implant strain under axial load. J Int Oral Health.

[16] Melo Filho AB, Tribst JPM Ramos NC, Luz JN, Jardini MAN, Borges ALS, Santamaria MP (2019). Failure probability, stress distribution and fracture analysis of experimental screw for micro conical abutment. Braz Dent J.

[17] Applegate OC (1965). Essentials ofremovablepartialdentureprosthesis. 3 ed.

[18] Costa VLS, Tribst JPM, Uemura ES, de Morais DC, Borges ALS (2018). Influence of thickness and incisal extension of indirect veneers on the biomechanical behavior of maxillary canine teeth. Restor Dent Endod.

[19] Arnold C, Hey J, Setz JM, Boeckler AF, Schweyen R (2018). Retention force of removable partial dentures with different double crowns. Clin Oral Investig.

[20] Kemmoku D (2012). BioCAD techniques: Example on maxilla for rapid expansion simulation.

[21] Dal Piva AO, Tribst JP, Borges AL, de Melo RM, Bottino MA (2019). Influence of substrate design for in vitro mechanical testing. J Clin Exp Dent.

[22] Sousa MP, Tribst JPM, de Oliveira Dal Piva AM, Borges ALS, de Oliveira S, da Cruz PC (2019). Capacity to maintain placement torque at removal, single load-to-failure, and stress concentration of straight and angled abutments. Int J Periodontics Restorative Dent.

[23] Al Jabbari YS (2014). Physico-mechanical properties and prosthodontic applications of Co-Cr dental alloys: a review of the literature. J Adv Prosthodont.

[24] Fujimoto T, Niimi A, Murakami I, Ueda M (1998). Use of new magnetic attachments for implant-supported overdentures. J Oral Implantol.

[25] Chun HJ, Park DN, Han CH, Heo SJ, Heo MS, Koak JY (2005). Stress distributions in maxillary bone surrounding overdenture implants with different overdenture attachments. J Oral Rehabil.

[26] Shahmiri RA, Atieh MA (2010). Mandibular Kennedy Class I implant-tooth-borne removable partial denture: a systematic review. J Oral Rehabil.

[27] Wismeijer D, Tawse-Smith A, Payne AG (2013). Multicentre prospective evaluation of implant-assisted mandibular bilateral distal extension removable partial dentures: patient satisfaction. Clin Oral Implants Res.

[28] de Freitas RF, de Carvalho Dias K, da Fonte Porto Carreiro A, Barbosa GA, Ferreira MA (2012). Mandibular implant-supported removable partial denture with distal extension: a systematic review. J Oral Rehabil.

[29] Matsudate Y, Yoda N, Nanba M, Ogawa T, Sasaki K (2016). Load distribution on abutment tooth, implant and residual ridge with distal-extension implant-supported removable partial denture. J Prosthodont Res.

[30] Campos CH, Gonçalves TM, Garcia RC (2015). Implant-supported removable partial denture improves the quality of life of patients with extreme tooth loss. Braz Dent J.

[31] Trakas T, Michalakis K, Kang K, Hirayama H (2006). Attachment systems for implant retained overdentures: a literature review. Implant Dent.

[32] Mitrani R, Brudvik JS, Phillips KM (2003). Posterior implants for distal extension removable prostheses: a retrospective study. Int J Periodontics Restorative Dent.

[33] Niinomi M (1998). Mechanical properties of biomedical titanium alloys. Elsevier.

